# Current stewardship practices in invasion biology limit the value and secondary use of genomic data

**DOI:** 10.1111/1755-0998.13858

**Published:** 2023-08-30

**Authors:** Amy L. Vaughan, Elahe Parvizi, Paige Matheson, Angela McGaughran, Manpreet K. Dhami

**Affiliations:** ^1^ Biocontrol & Molecular Ecology Manaaki Whenua Landcare Research Lincoln New Zealand; ^2^ Te Aka Mātuatua/School of Science University of Waikato Hamilton New Zealand

**Keywords:** biological invasion, invasion genomics, metadata, reference genomes, sequencing

## Abstract

Invasive species threaten native biota, putting fragile ecosystems at risk and having a large‐scale impact on primary industries. Growing trade networks and the popularity of personal travel make incursions a more frequent risk, one only compounded by global climate change. With increasing publication of whole‐genome sequences lies an opportunity for cross‐species assessment of invasive potential. However, the degree to which published sequences are accompanied by satisfactory spatiotemporal data is unclear. We assessed the metadata associated with 199 whole‐genome assemblies of 89 invasive terrestrial invertebrate species and found that only 38% of these were derived from field‐collected samples. Seventy‐six assemblies (38%) reported an ‘undescribed’ sample origin and, while further examination of associated literature closed this gap to 23.6%, an absence of spatial data remained for 47 of the total assemblies. Of the 76 assemblies that were ultimately determined to be field‐collected, associated metadata relevant for invasion studies was predominantly lacking: only 35% (27 assemblies) provided granular location data, and 33% (*n* = 25) lacked sufficient collection date information. Our results support recent calls for standardized metadata in genome sequencing data submissions, highlighting the impact of missing metadata on current research in invasion biology (and likely other fields). Notably, large‐scale consortia tended to provide the most complete metadata submissions in our analysis—such cross‐institutional collaborations can foster a culture of increased adherence to improved metadata submission standards and a standard of metadata stewardship that enables reuse of genomes in invasion science.

## 1 INTRODUCTION

As global trade networks expand and lifestyle‐associated travel continues to rise, so does the risk associated with biological incursions, where species establish populations outside their native range, often causing negative economic and ecological impacts (Hulme, 2021; Latombe et al., 2022; Seebens et al., 2018). The frequency and success of these incursions are only expected to increase (e.g. established alien species per continent are predicted to rise by 36% from 2005 to 2050; Seebens et al., 2021) as rising temperatures driven by anthropogenic climate change act to narrow the climatic gap between tropical and temperate regions (Gutierrez et al., 2021). Of the global pests routinely transported via human‐mediated or unaided pathways (as defined by Hulme et al., 2008), perhaps the most pervasive are invertebrates, with an estimated global cost exceeding US$700 billion from 1960 to 2020 primarily due to resource loss and direct damage (Renault et al., 2022). Global health is also affected by the zoonotic vectoring of insect‐borne diseases (Abubakr et al., 2022; Zhang et al., 2022), with implications for endemic biodiversity in natural habitats (Gentili et al., 2021).

As sequencing technologies have become more accessible, a surge in whole‐genome sequencing (WGS) and population genomic studies of invasive species has followed (Matheson & McGaughran, 2022). Collectively, these data hold the promise of disentangling the genomic mechanisms of invasion success. In the case of invasion genomics (the study of the genome within the context of biological invasion), WGS data are demonstrating emerging trends in invasive populations that are valuable for potential mitigation and control strategies. For example, genes associated with environmental adaptation (e.g. insecticide resistance and olfaction) have been identified in a range of unrelated invasive insects, such as the navel orangeworm *Amyelois transitella* (Pyralidae; Calla et al., 2020), the brown marmorated stink bug *Halyomorpha halys* (Hemiptera; Parvizi et al., 2023) and the fruit fly *Drosophila suzukii* (Diptera; Durkin et al., 2021). Rapid evolution in translocated populations, underpinned by either abiotic (such as climatic gradients in *Drosophila melanogaster*, Behrman et al., 2018); and across beetle families in Australia, (Wardhaugh et al., 2018) or biotic factors (e.g. new predator–prey interactions; Siepielski & Beaulieu, 2017) can be detected using invasion genomic approaches (North et al., 2021; Runquist et al., 2020). A recent study by Lukicheva and Mardulyn (2021) also identified evidence of asymmetric introgression in ~2% of the genome of an invasive leaf beetle, a low enough frequency to be undetectable via reduced representation sequencing. Thus, WGS is already beginning to elucidate intrinsic mechanisms that underpin an organism's ability to invade, and previous reviews have discussed the potential of this technology in invasion biology (McCartney et al., 2019; Rius et al., 2015). When inferring invasion events, rare variant single nucleotide polymorphisms (SNPs) or those unrepresented in a reference genome may not be identified in a low coverage genome, and inference of biologically important variants may be lost. Therefore, depending on the biological question, high sequencing depth and/or whole‐genome sequencing (versus reduced representation) data may be required to obtain an accurate representation of rare variant prevalence in populations (North et al., 2021).

To allow for reproducible experimentation, accurate and comprehensive metadata are required alongside published sequencing data. In the context of biological invasions, such spatiotemporal data are crucial for tracing incursion pathways (Bradhurst et al., 2021), whilst knowledge of host species may promote the study of reciprocal evolution in antagonistic species in host–parasite interactions (Beaurepaire et al., 2019). Such metadata can also extend the use of the sequencing data beyond a project's initial intent. Global databases, such as GenBank (https://www.ncbi.nlm.nih.gov/genbank/), require minimum metadata standards to be met at the time of WGS data submission, including the addition of collection date, geographic location, tissue type and host/source. But this list is not exhaustive and does not set requirements for the minimum standard of data provided (e.g. the precision of spatiotemporal data is not specified). In addition, factors such as geographic coordinates, sex and developmental stage are optional contributions. While optional metadata fields may remove some barriers to data submission, a lack of metadata can severely hamper the reusability of the submitted sequencing data (Tahsin et al., 2017). In the context of biological invasions, enriched metadata have enabled expansive research outside the original collection context, providing detail on expanded populations (Schmidt et al., 2023). High‐quality, complete spatiotemporal metadata records from museum samples have also allowed for a detailed analysis of divergent and parallel evolution during the invasion process (Stuart et al., 2022). Thus, without complete metadata records, the meaningful historic and biological context behind an invasion event can be lost.

To overcome the obstacles associated with metadata provision and data reuse, FAIR guiding principles of data stewardship state that data (and metadata) should be Findable, Accessible, Interoperable and Reusable (Wilkinson et al., 2016). In support of and adherence to FAIR guiding principles, recent genome sequencing projects and consortia—formed for example, with the purpose of sequencing all organisms (Lewin et al., 2022) or all life in the British Isles (Darwin Tree of Life project; Blaxter et al., 2022)—apply stringent data management policies in the recording of associated metadata. For instance, the Darwin Tree of Life project has recently proposed metadata standardization, encouraging other projects to adopt these strategies (Lawniczak et al., 2022). However, it is unclear how widely either individual researchers or consortia adhere to FAIR or other recommended data stewardship principles when capturing metadata associated with invasive species WGS experiments. To address this, we ask whether data stewardship practices in the field of invasion genomics may be limiting the value and secondary uses of genomic data.

We focus on data for terrestrial arthropods (Phylum: Arthropoda)—one of the most pervasive taxa in global incursions and among the most prevalent taxonomic groups sequenced. Collating publicly available WGS data for this group, we examine information supplied for key metadata categories with a particular focus on whether information relevant to large‐scale comparative genomic analyses (i.e. potentially applicable to broadscale insights into invasive potential) is available.

## 2 MATERIALS AND METHODS

A current list of invasive arthropod species with available reference genomes was compiled from the i5K Ag100Pest species list (Childers et al.,^2021^), the IUCN Global Invasive species database (GISD; Poorter et al., 2005), the IUCN 100 of the World's Worst Invasive Alien Species (WAS) list (Lowe et al., 2000), and directly from the literature. The Ag100Pest Initiative is a US‐specific project that aims to contribute arthropod genomes of 158 agricultural pest or potential pest species to the i5K 5000 arthropod genomes project (i5K Consortium, 2013) and Earth BioGenome Projects for cataloguing all eukaryotic diversity (Lewin et al., 2022). The WAS list comprises alien species from each taxonomic order, representing diverse taxonomic groups with large implications on human activity and species biodiversity, including 14 arthropod taxa. We searched the literature using PubMed, with search terms such as ‘invasive’, ‘WGS’ and ‘arthropod’, to identify any further invasive terrestrial arthropods for which WGS data were available. For each of the species on these combined lists (*n* = 240), invasive status was confirmed using wider literature searches and/or checks against the Invasive Species Compendium (www.cabi.org; CABI, 2023) in June 2023, resulting in a final list of 89 species with confirmed invasive status.

As the National Centre for Biotechnology Information (NCBI) is linked directly to the i5K project and is the largest compendium of genome data, we used NCBI to locate genome and associated metadata for these species. We first collected metadata from NCBI that was associated with the reference genome of each species, identifying ‘reference genome’ status based on its allocation as such within the NCBI database. We refer to this dataset of 89 species as the ‘reference genome’ dataset hereafter. Because reference genomes are often updated, thus several versions can exist for the same species, we also collected metadata for each subsequent genome assembly that was also available for any of these species in NCBI (‘assembly dataset’ hereafter). Between one and nine representative genome submissions were found for each of our 89 species, resulting in metadata for the assembly dataset for 199 whole genome assembly submissions (i.e. the ‘reference genome’ dataset is a subset of the ‘assembly’ dataset; Supplementary File S1). Alternative haplotypes and subsequent assemblies that represent whole‐genome resequencing population data (and not ‘reference genomes’) were not considered in the analysis.

### 2.1 Metadata compilation for assembly and reference genome datasets

Metadata was compiled from each assembly in the assembly and reference genome datasets, including spatiotemporal data, sex, tissue type and assembly level (chromosome, scaffold, or contig). Spatiotemporal data were collected at country, region, coordinate, year, month and date levels. Tissue type defining whole, sectional or pooled samples, as well as life stage was recorded, while sex was listed as either female or male. For those genomes that did not provide spatiotemporal data to classify origin as either wild collected or laboratory/colony reared, associated literature searches with the accession/BioProject number were used to further determine this, or a classification of unknown origin was recorded. The organization and country associated with each assembly submission was assessed to understand the distribution of invasive species research globally. A map of spatial level metadata was then generated using ArcMap 10.8.2. Frequency of submitting institutes was tallied, with ‘high income’ origin countries defined as those where gross national income per capita exceeds USD12,055 (www.worldeconomics.com/Regions/High‐Income‐Countries/). Accessibility of raw sequencing data was recorded by the presence of linked Sequence Read Archive (SRA) identifiers. Submission institute/country was also collected as described above. Finally, metadata was compared across all fields to determine overall data completeness for both the reference genome and assembly datasets.

### 2.2 Field‐collected subset analysis

Within invasion studies, field‐collected samples are the most valuable data asset, as they represent a genomic snapshot from within the native/expanded range at a specific time point that can be related to incursions. For this reason, the subset of assemblies from the assembly dataset that were identified as field‐collected was further analysed. Status as native/expanded was recorded based on the confirmed collection location, which was correlated with the known geographical home range of each species using the CABI database. The quantity of native/expanded range assemblies per species was then determined, as was the completeness of spatiotemporal data, the tissue type/sex, and submitting institute or consortia. Submission year was also compiled for this subset of field‐collected assemblies to investigate temporal metadata trends.

## 3 RESULTS

### 3.1 Current trends in invasive species genome sequencing

As of June 2023, 55 of 158 species (35%) in the AG100Pest list had a publicly available reference genome. Thirty‐four of these agricultural pests were further classified as ‘invasive’ using literature and the CABI database and, of these, 26 species had more than one assembly available on NCBI. Of the IUCN GISD list—when restricted to terrestrial arthropods (*n* = 86)—30 species (~34.9%) had publicly available reference genomes/assemblies. Meanwhile, of the 14 invasive insects included on the WAS list, nine (64%) had a publicly available reference genome and five of these had >1 available genome assembly. This resulted in a total dataset of 89 and 199 reference genomes and assemblies, respectively.

**FIGURE 1:**
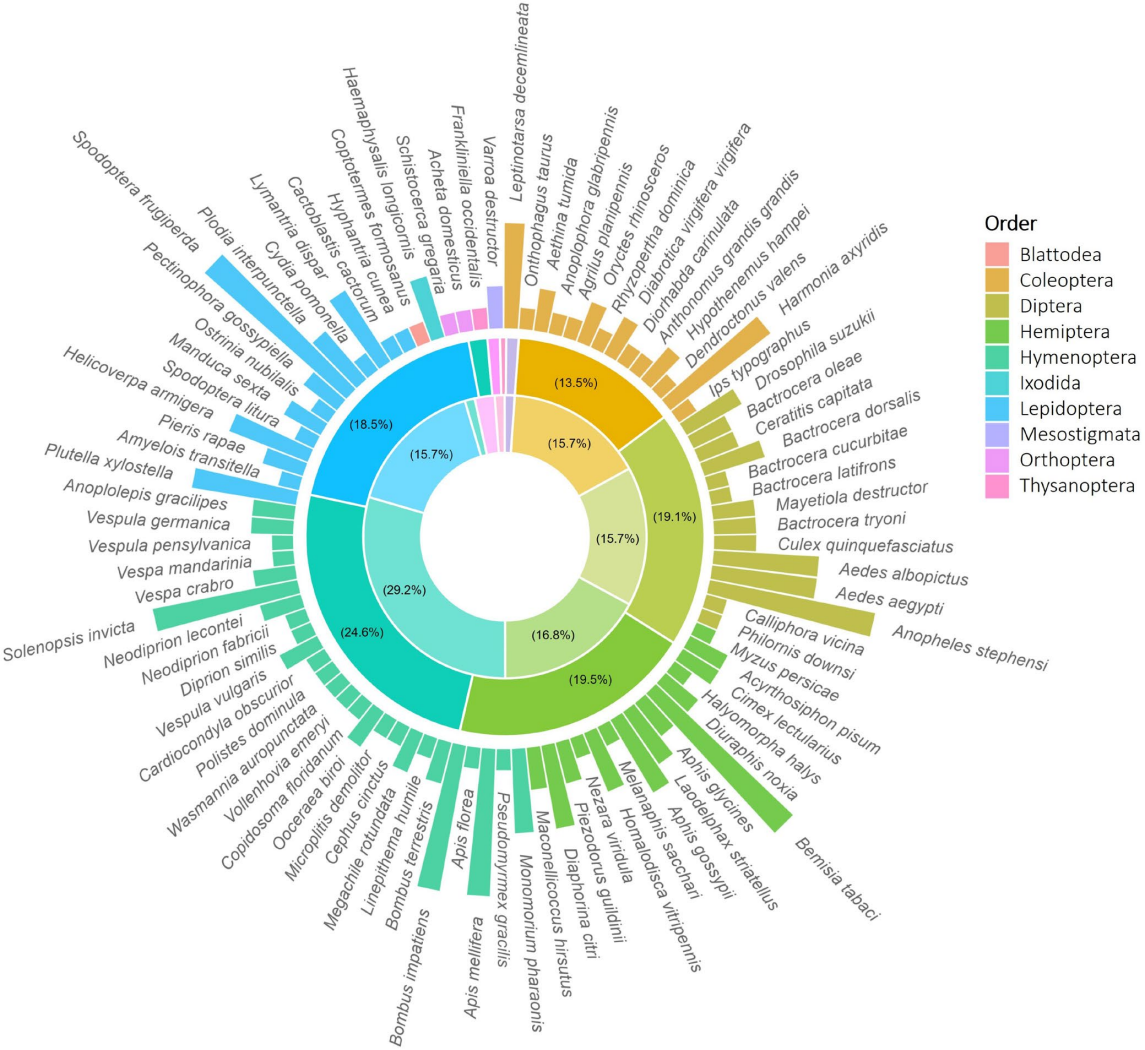
Genome assembly frequency and taxonomic coverage. Bars represent the relative frequency (*n*  = 1–9) of individual genomes identified within the NCBI repository for 89 terrestrial arthropods, with species coloured by taxonomic order according to the provided key; Inner plot shows the proportion of species comprising each taxonomic order between the reference genome (inner ring; *n* = 89) and assembly (outer ring; *n* = 199) datasets.

The NCBI and i5K database searches together identified 10 orders of terrestrial arthropods for which reference genomes were available, with the largest proportion of data belonging to Hymenoptera (29%), Diptera (15%) and Coleoptera (15%), and Blattodea and Thysanoptera least represented (~0.5%, Figure 1). Genome assemblies were distributed unequally across the 89 species, with some having a low frequency and others (typically of high risk of invasion and importance to current research within the field of invasive biology) having increased representation (e.g. *Spodoptera frugiperda*, *n* = 9; Figure 1) (Supplementary File S1).

High‐income countries dominated institutional representation in the assembly dataset, with the top four countries (USA = 76/199, UK = 22/199, Switzerland = 9/199, and France 6/199) contributing 64% of the available assemblies. Overall, the high‐income countries group accounted for ~68.8% of the assembly dataset (*n* = 137). Meanwhile, international consortia (such as i5K; *n* = 12) were responsible for 24 (12.61%) of the 199 assemblies.

**FIGURE 2:**
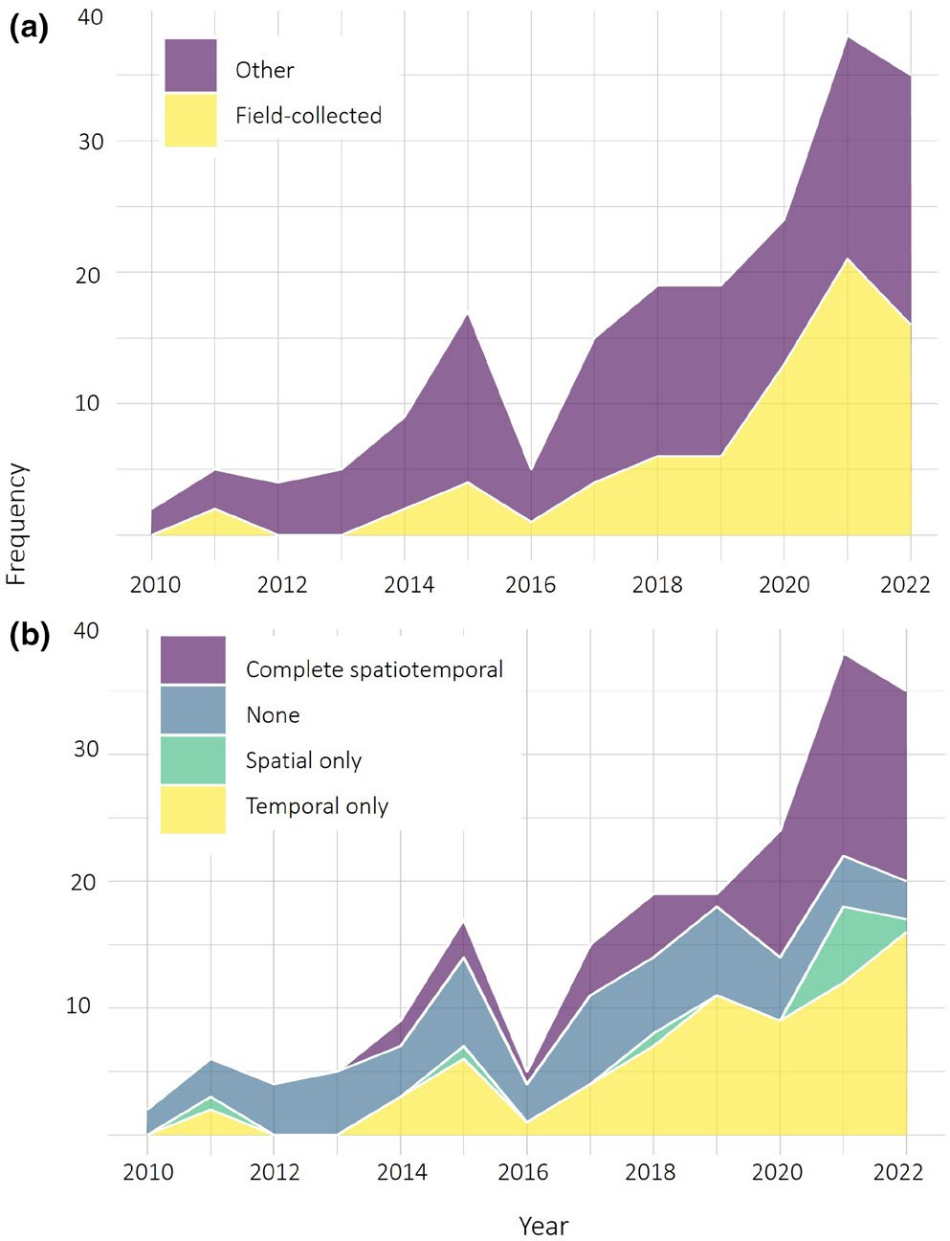
Frequency of genome assembly deposition to repositories and their relative spatiotemporal metadata entry completeness for invasive terrestrial arthropods between 2010–2022. (a) Data were separated into ‘other’—defined as the combination of lab, commercial, and unknown origins—and ‘field‐collected’ based on metadata associated with 199 assemblies, using submission date for each assembly as a classifier. (b) Completeness of spatiotemporal metadata in the assembly dataset by submission year, where ‘complete’ refers to spatial data that includes collection status (field, laboratory), provision of geographic location (to at least country), and temporal (to at least year of collection) information.

The number of deposited invasive species genome assemblies showed an increase with time, with almost half collected in the last 5 years and 19 assemblies deposited in 2018 and 2019 (Figure 2a). An increasing trajectory was observed in the completion of spatiotemporal metadata across the assessed time period (2010–2022), showing a general trend towards improvement in metadata curation and completion (Figure 2b).

### 3.2 Sample collection metadata is limited across reference and assembly datasets

**FIGURE 3:**
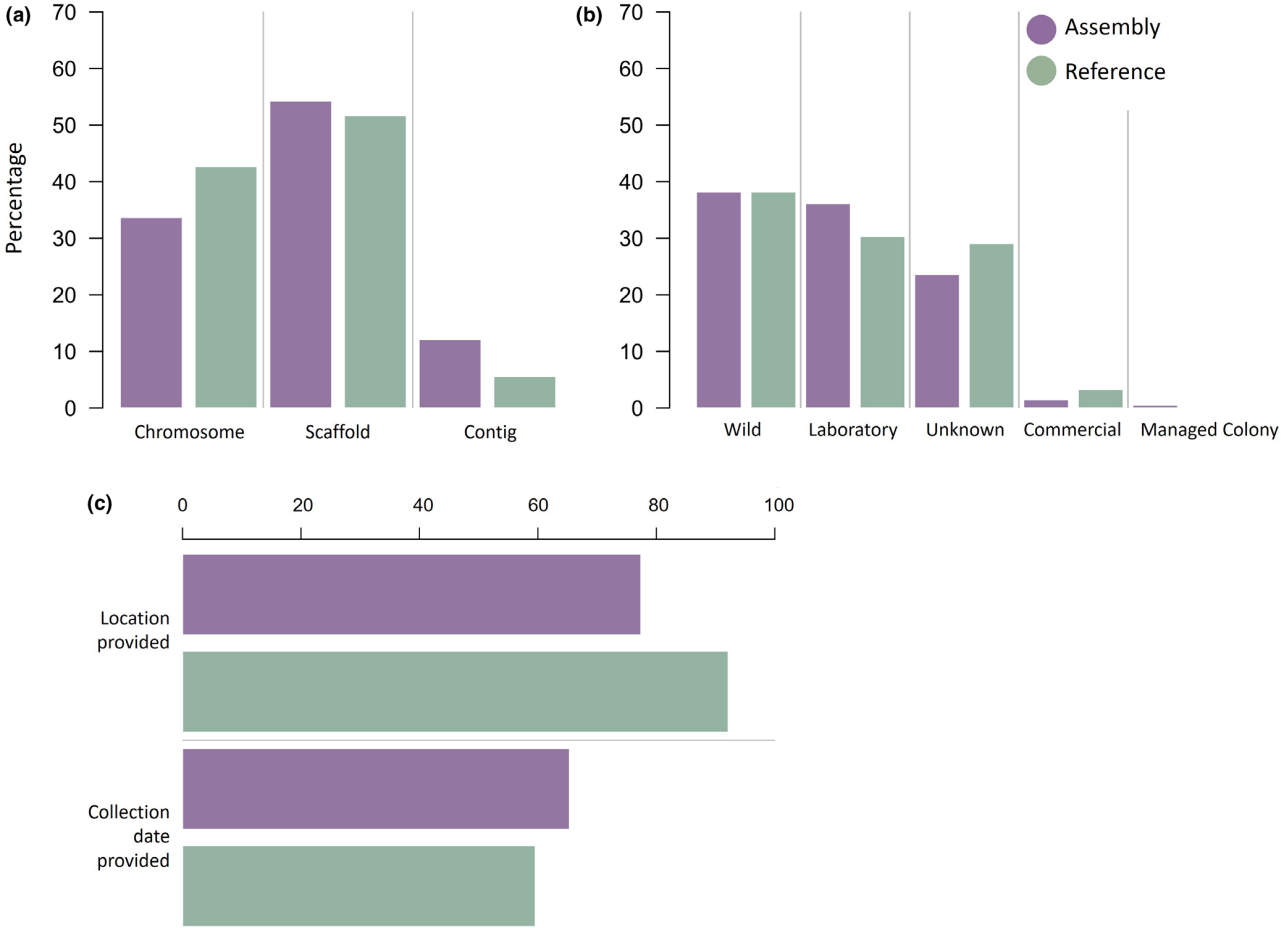
Metadata trends for reference genome (*n* = 89) and assembly (*n* = 199) datasets. (a) Assembly completeness (chromosome, scaffold, or contig level); (b) Sample origin (wild, laboratory, commercial, and managed colony); and (c) Completeness of spatiotemporal metadata (location and/or collection date provided).

Most of the 89 reference genomes were assembled to chromosome (42%) or scaffold level (51%), with few representations at contig level (5.6%). This contrasted with the assembly dataset, where 33% (*n* = 67) and 54% (*n* = 108) of assemblies had chromosome and scaffold level completeness, respectively (Figure 3a).

When identifying the origin of the reference genomes, 38% (*n* = 34) were field‐collected, 34% (*n* = 30) were derived from laboratory/commercial colonies and 28% (*n* = 25) had an unknown origin (Figure 3b). GPS coordinate level location information was provided for 25% (*n* = 22) of reference genomes overall, and 14 of these were from free‐living field‐collected organisms (Figure 3c). The frequency of entries with no associated collection date was high (40%, *n* = 36), though only one of these was found to be from a field‐collected organism. However, collection date specificity was not uniform, with 18 (20%) of those that did provide a sample collection date only providing the year of collection (Figure 3c).

Approximately 32% of the total assemblies (*n* = 64) were classified as field‐collected, 28% (*n* = 56) originated from laboratory/commercial colonies, and ~ 38% (*n* = 76) had unknown origin (Figure 3b). For the 76 assemblies that did not provide adequate metadata to determine sample origin, literature searches identified 12 additional assemblies derived from field‐collected organisms (raising the total to *n* = 76), while an additional 17 were determined to be from laboratory/commercial colonies (total increased to *n* = 75). Sample origin information could not be retrieved for the remaining 47 assemblies, thus, origin information was ultimately obtainable for 76.3% of the assembly dataset. Metadata for 45 assemblies (22.6%) did not provide specific sample collection location (e.g. GPS coordinates; country), while 69 assemblies (34.6%) did not provide collection year.

Analysis of further metadata fields found a high degree of missing data, with absent tissue type information for 27 (30%) of reference genomes and 65 (32%) of assemblies. Similarly, developmental stage information was missing for 38 (42%) reference genomes, and 65 (32.6%) assemblies, and sex was not provided for 33 (37%) and 99 (49.7%) of reference genome and assembly data, respectively. Finally, we found that 64% of assemblies had associated raw data that was submitted with an accessible SRA identifier.

### 3.3 Spatiotemporal origins of field‐collected samples remain elusive

Of the 76/199 total assemblies identified as field‐collected (including 12 assemblies which we added via literature review; see above), 39 (50.6%) were also classified as ‘reference genomes’ for their species, and 50 (56%) of the 89 total species included in this study were represented (Supplementary File S1).

Field‐collected assemblies had a higher proportion of data relating to tissue type compared with the complete assembly dataset, with 46 (60.5%) and 47 (61.8%) including information on the developmental stage and sex, respectively. The representation of complete chromosome level sequences was slightly higher in field‐collected assemblies versus the whole assembly dataset (34.2% vs. 33.7%) but lower than reference genomes (34.2% vs. 42.7%). Overall, ~70% of field‐collected assemblies were of scaffold level or higher.

**FIGURE 4:**
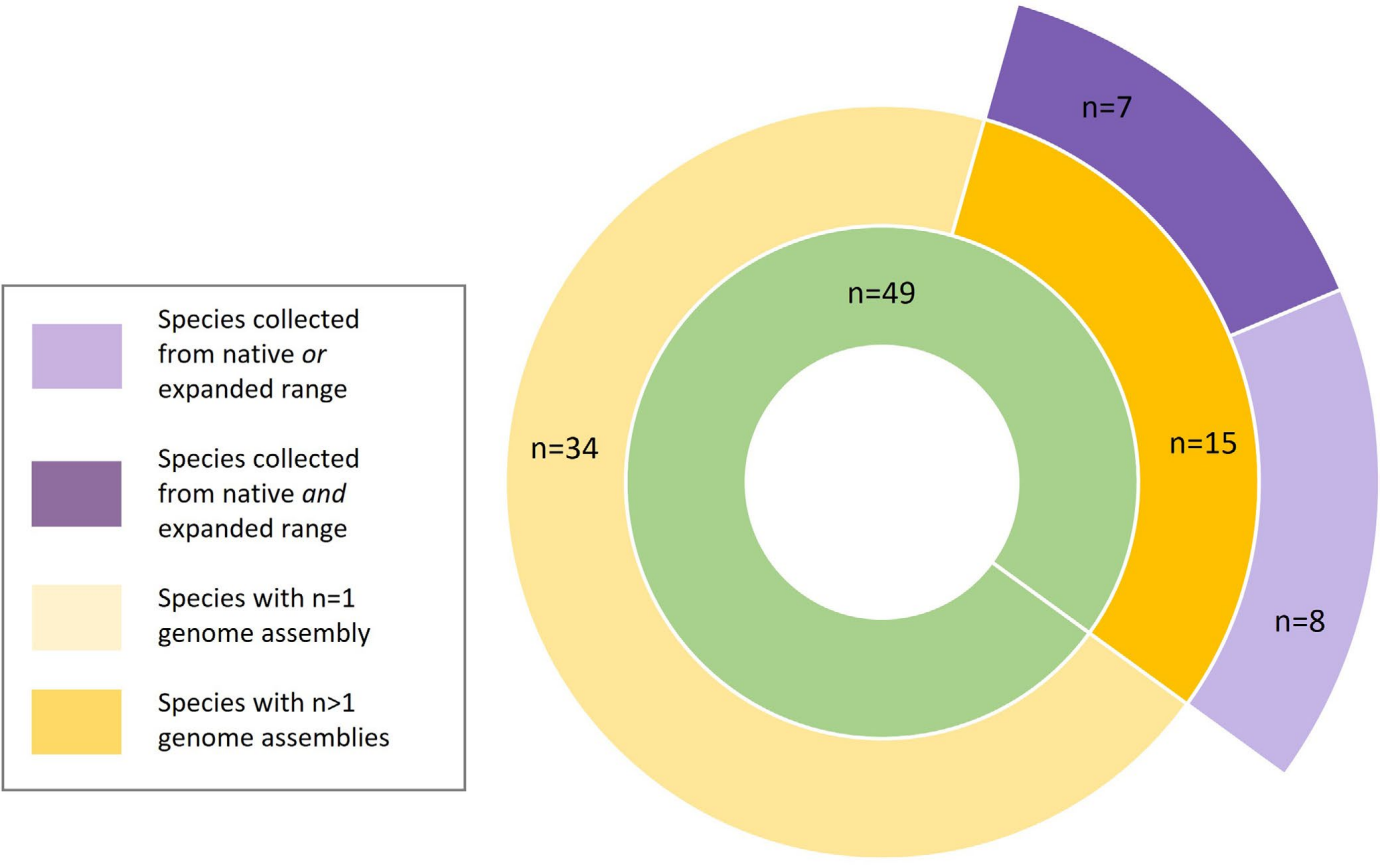
Metadata trends for field‐collected species assemblies. The proportion (from a total of *n* = 49; green inner circle) of species with field‐collected data, including those with single or multiple assemblies (shades of yellow), and those that included information on native or expanded range (shades of purple). Includes two assemblies of *Aphis gossypii*, where the origins of the species were unknown but were specified as field‐collected.

A slightly higher proportion of invasive species assemblies that came from field‐collected samples were collected from their native (*n* = 40, 51.9%) rather than expanded range (*n* = 33, 42.9%) (Figure 4). Without the addition of assemblies from the literature review, these numbers reduced further for expanded range assemblies (*n* = 26, 40%) though increased for native range assemblies (*n* = 35, 53.8%). For four assemblies with field‐collected status, native/expanded status could not be determined, as specific location was unknown, or the species was listed as feral.

**FIGURE 5:**
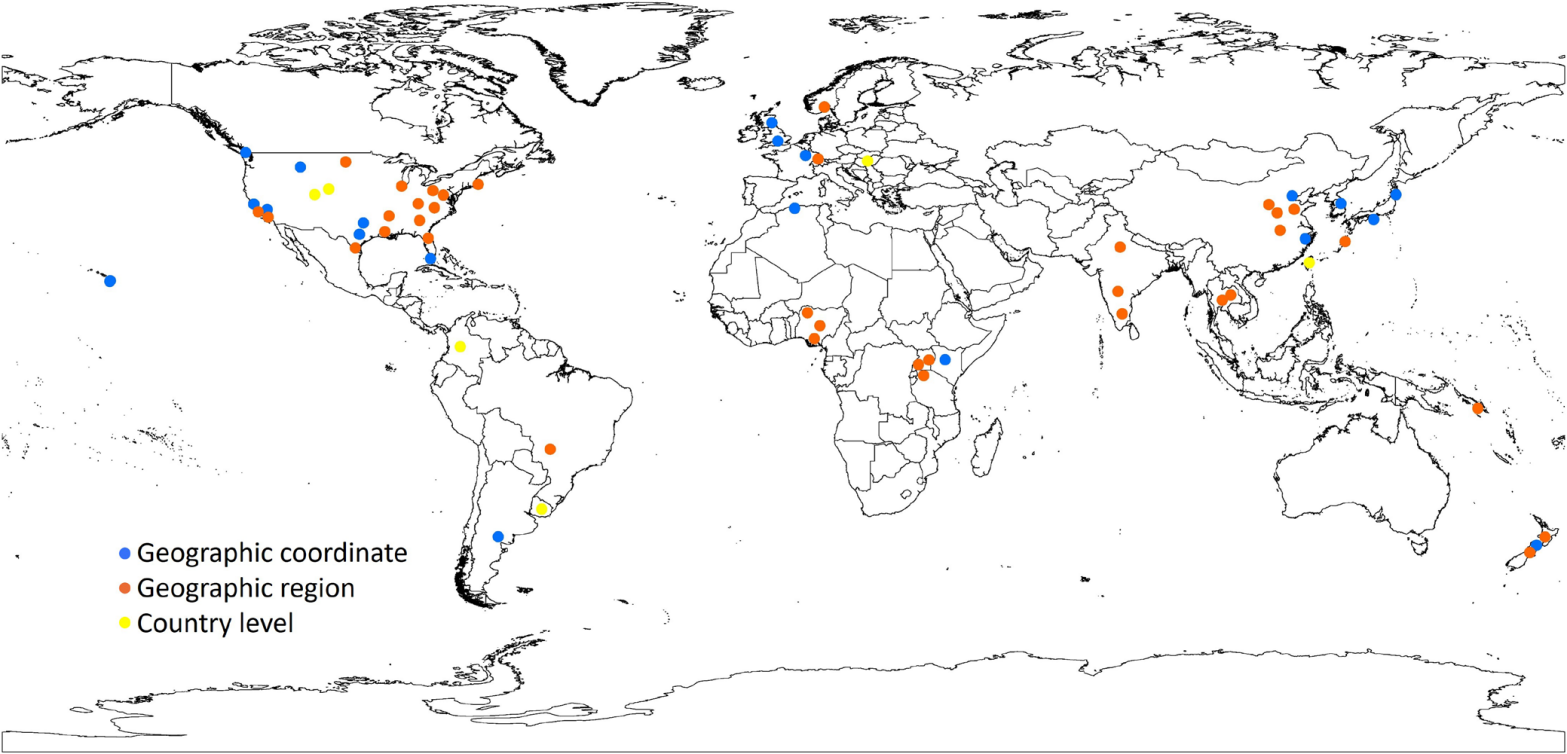
Level of geographic metadata provided for field‐collected sample assemblies. Sampling sites for field‐collected sample assemblies were reported at three different resolutions, as indicated by the provide key.

Specific location data for most of these assemblies were restricted to region or city level, with location data provided only at the country level for eight (10%) of assemblies. Only 35.5% provided GPS coordinates, including six (26%) from the Wellcome Sanger Institute. Of the 76 field‐collected assemblies, 58% (*n* = 44) were collected from high‐income group countries (Figure 5).

Seven species had assemblies from field‐collected specimens that were collected from both the native and expanded ranges (Table 1). These included six insects and one arachnid species (glassy‐winged sharpshooter; *Homalodisca vitripennis*: Hemiptera), common wasp (*Vespula vulgaris*: Hymenoptera), small white butterfly (*Pieris rapae*: Lepidoptera), harlequin ladybird (*Harmonia axyridis*: Coleoptera), asian citrus psyllid (*Diaphorina citri*: Hemiptera), european wasp (*Vespula germanica*: Hymenoptera) and asian longhorned tick (*Haemaphysalis longicornis*: Ixodida; added via literature review), representing just 7.8% of the complete list of 89 species in our analysis, and 7.5% of the genome assemblies.

**TABLE 1:** Seven species for which both native and expanded range assemblies were available.

Order	Species	Common name	Range	GenBank accession	Reference	Useful insights from metadata and potential future uses
Hemiptera	*Homalodisca vitripennis*	Glassy‐winged sharpshooter	Native: Texas, United States	GCA_021130785.2	(Li et al., 2022)	
			Expanded: California, United States	GCA_019364655.1	(Ettinger et al., 2021)	Confirmation of reference genome from invasive populations, future studies on population structure and development of nonbiological controls
Hymenoptera	*Vespula vulgaris*	Common wasp	Native: Wytham Great Wood, United Kingdom	GCA_905475345.1	(Crowley, 2022)	
			Expanded: Pelorus, New Zealand	GCA_014466185.1	(Harrop et al., 2020)	Provided genomic targets for applied management. Comparisons between native/expanded populations would need to eliminate targets that can harm populations within the native range.
Lepidoptera	*Pieris rapae*	Small white butterfly	Native: West Linton, Scotland, United Kingdom	GCA_905147795.1	(Lohse et al., 2021)	Further insights of the species ecology by sequencing from the native range after Shen et al. (2016) sequenced individuals from an invasive population.
			Expanded: Texas, United States	GCA_001856805.1	(Shen et al., 2016)	
Ixodida	*Haemaphysalis longicornis*	Asian longhorned tick	Native: Shandong, China	GCA_013339765.2	(Jia et al., 2020)	
			Expanded: New Zealand	GCA_008122185.1	(Guerrero et al., 2019)	Study of pathogenesis related genes from a population that solely utilizes parthenogenetic reproduction.
Coleoptera	*Harmonia axyridis*	Harlequin ladybird	Native: Japan	GCA_003402655.1	—	
			Expanded: Wytham Great Wood, United Kingdom	GCA_914767665.1	(Boyes & Crowley, 2021)	
Hemiptera	*Diaphorina citri*	Asian citrus psyllid	Native: Taiwan	GCA_024506325.2	(Carlson et al., 2022)	Creation of three genomes from the same BioProject with the goal to compare between native and expanded ranges, demonstrating congruent results to known invasion histories.
			Expanded: Uruguay	GCA_024506275.2	(Carlson et al., 2022)	Temporal data provided context of population to known invasion history.
			Expanded: Los Angeles and California, United States	GCA_024506315.2	(Carlson et al., 2022)	Temporal data provided context of population to known invasion history.
Hymenoptera	*Vespula germanica*	European wasp	Native: Wytham Rough Common, United Kingdom	GCA_905340365.1	(Crowley, 2022)	
			Expanded: Lincoln, New Zealand	GCA_014466195.1	(Harrop et al., 2020)	As with the common wasp, any method of targeted management would need to eliminate genomic targets that can harm populations within the native range.

## 4 DISCUSSION

With the rise in accessibility of next‐generation sequencing technologies in the last decade, research into the genomics of invasive species is burgeoning. Yet, as the number of reference genomes and genome assemblies has increased, we found an increasing tendency for incomplete metadata associated with submissions, limiting the usability of these genomic resources for the study of mechanisms that underpin invasion success.

Only 38.2% of reference genomes, and 38.1% of assemblies in our respective datasets contained enough metadata to be comprehensively classified as field‐collected. Yet, undertaking large‐scale, comprehensive comparative genomic analysis to identify signals of invasiveness requires genome assemblies of species collected from their native and expanded ranges (Dematteis et al., 2020; North et al., 2021; Turner et al., 2021) to identify rare variants that may not be present within a single reference genome (e.g. following post‐invasion adaptation). For example, Carlson et al. (2022) sequenced three high‐quality chromosome scale assemblies of *Diaphorina citri* (from two expanded and one native range individuals) and found that the native range strain was more similar to one of the expanded range strains in a manner congruent with the known invasion history. Such studies cement the value of using high‐quality chromosome level genomes from both invaded and native ranges in invasion studies. The absence of environmental context for most invasive insects effectively excludes these genomes/assemblies from future analysis (without additional sequencing) where the aim is to understand adaptation in the invaded range. For example, studies of *D. suzukii* demonstrate that the genome content of laboratory colonies does not necessarily mirror that of free‐living individuals, with samples derived from laboratory populations in Japan and Hawai'i segregating in population structure analyses (Lewald et al., 2021). Similarly, a study by Lainhart et al. (2015) on laboratory isolates of the mosquito *Anopheles darlingi* after 21 generations showed the effects of genetic bottlenecks within populations, including decreased heterozygosity compared with the founder population. Studies like these suggest that the genetic drift commonly observed in long‐term laboratory populations would make them unsuitable for large‐scale comparisons between invasive and non‐invasive species, as well as potentially for intraspecific comparisons between native and expanded ranges. Nevertheless, our data indicate that invasive species genomic data are commonly derived from laboratory and commercial lines (30/89 reference genomes; 75/199 assemblies).

In our dataset, 28% of reference genomes and 23.6% of genome assemblies had an unknown or unlisted origin. This compares with a large‐scale analysis by Toczydlowski et al. (2021), which showed that spatiotemporal metadata was present in 51% of ‘wild’ datasets of eukaryote species (and just 39% where location data was specified as GPS coordinates). Our findings likely also translate to other species, though the number of field‐collected samples with geotagged locations is likely to be much higher in some studies (e.g. those focussed on population genomics). Original study objectives are therefore likely to be a limiting factor in the reuse of data, and generation of ‘intention free’ data that includes all available metadata would have far‐reaching benefits for invasion biology and the wider genomics research community.

Overall, 40% of the 89 species, and 35% of the 199 assemblies also lacked a collection date. An important aspect to invasion biology are comparisons of historic and contemporary data to identify key genomic changes over time. For example, findings of parallel selection across historic and contemporary samples of *Sturnus vulgaris* in the UK and Australia suggest occasional convergent responses in both the native and expanded range, demonstrating the value of studying historic data (Stuart et al., 2022, 2023). Historical collections (e.g. museums, herbaria) are commonly used to address such questions, where samples have not been previously collected and retained for this purpose but the provision of relevant temporal data would allow the future reuse of contemporary samples in time series studies. A crucial area of focus lies in reconstructing invasion histories and examining genomic and genetic connections between native and expanded range populations. Reconstruction of invasion histories facilitates applied strategies for prevention and mediation of biological invasions, though the completion of comprehensive spatiotemporal metadata is crucial for facilitating such transgenerational analysis (Estoup & Guillemaud, 2010). Completion of collection date or location metadata can allow researchers to trace gene flow among populations, potentially identifying the source of initial bridgehead populations (Vallejo‐Marín et al., 2021), and/or infer whether single or sequential incursion events occurred to establish the invasive population(s) (Blumenfeld et al., 2021; Puckett et al., 2020). Completion of comprehensive metadata therefore allows for a better understanding of invasion dynamics.

Contrary to trends in spatiotemporal data, developmental stage and sex were well‐represented in the reference genome and assembly datasets. Ninety‐five percent of assembly data included the development stage, though only 57% of the reference genomes followed suit. Meanwhile 50% and 62% of the assembly and reference genome datasets stated the sex of the organism, respectively. Demonstrating the importance of these data fields, functional transcriptome analysis of adult female *Drosophila* spp., including the highly invasive *D. suzukii*, allowed a qualitative overview of gene expression across ovipositor shapes, the mechanism by which female *D. suzukii* can expand its host range (Crava et al., 2020). Thus, incorporating physiologically meaningful information into metadata fields could improve understanding of how genomic traits underpin physiological flexibility in invasive species. While these additional fields of metadata may have a less direct relevance for some applications (e.g. comparative genomics), they are important for the purposes of future reuse of data, and to adhere to the FAIR principle of interoperability (i.e. to set baseline standards of metadata collection regardless of experimental intent).

Seven species in our analysis had both native and expanded range representation, with temporal (93.4%), developmental stage (93.4%) and sex (80%) metadata provided (Boyes & Crowley, 2021; Carlson et al., 2022; Crowley, 2022; Ettinger et al., 2021; Guerrero et al., 2019; Harrop et al., 2020; Jia et al., 2020; Li et al., 2022; Lohse et al., 2021; Shen et al., 2016). Interestingly, none of these studies were conducted within a principle invasion context, though Ettinger et al. (2021) acknowledged that the sequencing of the invasive representative was a critical resource for management strategies in the glassy‐winged sharpshooter, and this sentiment was reiterated by Harrop et al. (2020) for the common wasp. Of the studies conducted on these eight species (15 genome assemblies), 10 were within the last 5 years, emphasizing the recently improved accessibility of high‐throughput sequencing and the promising trend of improving metadata quality we identified for the insects in our dataset. In particular, the number of assemblies that came from samples that were classified as field‐collected increased from 29 to 65 between 2016 and 2021. A recent meta‐analysis confirmed this trend, showing a 13.5% yearly decrease in the probability of retrieving missing metadata across multiple sequencing projects—indicating that new metadata is more likely to be complete (Crandall et al., 2023). Indeed, ten Hoopen et al. (2016) demonstrated that post‐hoc manual sample curation was laborious and difficult to implement on a large scale. FAIR‐adhering initiatives, such as the Genomic Observatories Metadatabase (GEOME; Deck et al., 2017), are a practical solution for authors to make metadata of previously published genomic and genetic datasets available retrospectively.

Perhaps unsurprisingly, the most consistent and thorough metadata submissions in our analysis came from the Wellcome Sanger Institute (Blaxter et al., 2022), where the code of practice for sampling specifically aims for the maximum reuse of each genome assembly, following FAIR principles (Wilkinson et al., 2016). These project submissions included geographic coordinates (and altitude in m), specific collection dates, and detailed sample notes on developmental stage and tissue type as per the Darwin Tree of Life's sample manifest (www.github.com/darwintreeoflife/metadata/). In invasion genomics, these metadata fields are especially useful in allowing users to determine the range type and invasion status of the population the individual was sampled from, as demonstrated in recent population genomic studies on invasive species (e.g. Parvizi et al., 2023; Schmidt et al., 2021).

As research questions develop and technology continues to evolve, the ability to use genomic tools to answer fundamental questions in invasion biology, such as identifying the genes responsible for an organism's mechanistic ability to invade, will increase (Gill et al., 2021). Our study supports calls for standardized metadata in genome assembly data submissions (Díaz et al., 2020; Toczydlowski et al., 2021) to facilitate this exciting future work. In particular, we have demonstrated that current metadata stewardship falls short of what is required for large‐scale comparative studies in invasion biology. We suggest four recommendations that would improve metadata curation:

1. Mandatory minimum metadata requirements for spatiotemporal data, ideally to include decimal latitude/longitude, environmental medium/habitat, and date to mm/yyyy level. Current standards utilized by institutes such as Wellcome Sanger have stringent, comprehensive metadata requirements and would be appropriate for adoption by the wider community.
2. Standardized field codes for metadata input, with date and decimal latitude/longitude to be in uniform field descriptions, in addition to drop‐down selections for wild collected, laboratory or colony strain origin states. Optional strain number, source or voucher accession number could also be valuable.
3. Greater inter‐institute collaboration in defining baseline standards for metadata curation, with demonstrable and uniform adherence to FAIR principles. Establishment of working groups or active discussions at conferences and workshops to define these baselines would be a good starting point for establishing widespread metadata standards.
4. Authors should retrospectively enrich metadata curation associated with publicly available genome datasets where possible, using open access infrastructure such as GEOME, or any other FAIR‐compliant open repositories.

While these improvements would have far‐reaching benefits, in invasion research they would specifically allow for determination of a samples' status regarding field versus laboratory collection, pre‐ versus post‐invasion, and native versus expanded range, facilitating broader comparative research and exciting new insights into the role of genome architecture in biological invasion.

## AUTHOR CONTRIBUTIONS

All authors participated in study conceptualisation. ALV analysed the data and wrote the manuscript draft. All authors edited the manuscript and approved the final version.

## CONFLICT OF INTEREST STATEMENT

The authors declare no conflicts of interest.

## BENEFIT‐SHARING STATEMENT

This study promotes international scientific partnership in the development of metadata standards for facilitation of broader future studies. Our research addresses limitations in the recording of whole genome sequencing metadata for invasive species and encourages the broader scientific community to initiate data standards conversations for the benefit of future studies.

## Supporting information


Data S1

## Data Availability

No new genomic data were generated during the course of this study. All accession numbers and related SRA and BioProject numbers for each reference genome and assembly can be found in Supplementary File S1.
